# *Radix Rehmanniae* Extract Ameliorates Experimental Autoimmune Encephalomyelitis by Suppressing Macrophage-Derived Nitrative Damage

**DOI:** 10.3389/fphys.2018.00864

**Published:** 2018-07-20

**Authors:** Wenting Li, Hao Wu, Chong Gao, Dan Yang, Depo Yang, Jiangang Shen

**Affiliations:** ^1^LKS Faculty of Medicine, School of Chinese Medicine, The University of Hong Kong, Hong Kong, Hong Kong; ^2^Department of Chemistry, The University of Hong Kong, Hong Kong, Hong Kong; ^3^School of Pharmaceutical Sciences, Sun Yat-sen University, Guangzhou, China

**Keywords:** *Radix Rehmanniae*, multiple sclerosis, macrophage, nitrative damage, (NF-κB) signaling pathway

## Abstract

Multiple sclerosis (MS) is a neuroinflammatory disease in central nervous system (CNS) without effective treatment or medication yet. With high prevalence of MS patients worldwide and poor therapeutic outcome, seeking novel therapeutic strategy for MS is timely important. *Radix Rehmanniae* (RR), a typical Chinese Medicinal herb, has been used for neuroinflammatory diseases in Traditional Chinese Medicine for centuries. However, scientific evidence and underlying mechanisms of RR for MS are unclear. In this study, we tested the hypothesis that RR could attenuate the progress and severity of MS via suppressing macrophage-derived nitrative damage and inflammation by using experimental autoimmune encephalomyelitis (EAE) model for mimicking MS pathology. The results showed the RR treatment effectively ameliorated clinical disease severity, inhibited inflammation/demyelination in spinal cord, and alleviated CNS infiltration of encephalitogenic T cells and activated macrophages. Meanwhile, RR possessed bioactivities of scavenging ONOO^−^ and reducing the expression of iNOS and NADPH oxidases in the spinal cords of the EAE mice. Furthermore, RR treatment suppressed nuclear factor-κB (NF-κB) signaling pathway in the splenocytes of EAE mice. The *in vitro* experiments on macrophages and neuronal cells exerted consistent results with the *in vivo* animal experiments. Taken together, we conclude that *Radix Rehmanniae* extract has therapeutic values for ameliorating EAE/MS pathological process and disease severity and its underlying mechanisms are associated with anti-inflammation and inhibiting macrophage-derived nitrative damages. Further study could yield novel promising therapeutic agent for multiple sclerosis.

## Introduction

Multiple sclerosis (MS) is an inflammatory auto-immune disease characterized by focal demyelination, axonal and neuronal damage in central nerve systems (CNS) (Thompson et al., 2010). Currently, immunomodulation and immunosuppression are the major therapeutic strategies for MS but carry severe side effects. With the prevalence of 2.5 million MS patients worldwide and poor therapeutic outcome, the majority of MS patients eventually develop into handicap. Seeking novel therapeutic strategy for MS is timely important. Experimental autoimmune encephalomyelitis (EAE) is a widely adopted animal model mimicking the key features of MS, including CNS-directed leukocyte infiltrations and inflammatory microenvironment induction, which destroy CNS structures and result in progressive paralysis (Lassmann and van Horssen, 2011). EAE animal model provides a reliable tool not only for understanding the mechanisms of MS but also for drug discovery.

Free radicals, including reactive oxygen species (ROS) and reactive nitrogen species (RNS), are intimately associated with MS pathogenesis. Oxidative stress-mediated CNS damages have been found both in both MS patients as well as EAE animal model (Cross et al., 1997; Smith et al., 1999). Both oligodendrocytes and neurons are highly vulnerable to oxidative/nitrative damage (Jack et al., 2007; Bishop et al., 2009). Excessive ROS/RNS derived from macrophage triggers oxidative damages, aggravates demyelination, axonal degradation, and neuronal cell death (Dunham et al., 2017). As a representative RNS, peroxynitrite (ONOO^−^) is rapidly produced by the reaction of superoxide (${\text{O}}_{2}^{•-}$) and nitric oxide (NO). ONOO^−^ exerts strongly membrane penetrability and highly cytotoxicity to CNS. Both human adult CNS-derived oligodendrocytes and motor neurons are highly susceptible to ONOO^−^-mediated injury (Li et al., 2011; Nikić et al., 2011). Increased 3-nitrityrosine (3-NT), a footprint marker of ONOO^−^, was identified in the oligodendrocytes of the MS samples featured oligodendrocytes death (Jack et al., 2007). Acute and chronic relapsing pathogenesis of EAE was ameliorated by the treatment of 5,10,15,20-tetrakis (4-sulfonatophenyl) porphyrinatoiron (III) chloride (FeTPPS), a representative peroxynitrite decomposition catalyst (PDC) (Bolton et al., 2008). Furthermore, MS patients had low level of uric acid, an ONOO^−^ scavenger, in serum and CSF associated with disease activity or treatment response (Dujmovic et al., 2009). Therefore, targeting ONOO^−^ could be an important therapeutic strategy for EAE or MS.

Traditional Chinese medicine (TCM) has been used for neurodegenerative diseases including MS (Liu et al., 2012). *Radix Rehmanniae* (RR), is one of the most frequent used herbal items in TCM formulas for MS patients (Song et al., 2017). RR exerts various bioactivities such as anti-osteoporotic (Oh et al., 2003), anti-inflammatory (Kim et al., 1999; Lau et al., 2009), immunomodulatory (Kim et al., 1998; Sung et al., 2011) and neuroprotective effects (Yu et al., 2006; Lee et al., 2011). The neuroprotective effects of RR could be attributed to the properties of antioxidant and anti-inflammations (Tian et al., 2006). RR attenuated the cisplatin-induced damage in HEI-OC1 auditory cells and the underlying mechanisms could be attributed to its antioxidant properties by inhibiting lipid peroxidation and scavenging free radicals, including superoxide radical, hydroxyl radical, and hydrogen peroxide (Yu et al., 2006). However, direct evidence about the neuroprotective effects of RR for MS or EAE is still lack. In the present study, we tested the hypothesis that RR could attenuate neuroinflammation and demyelination in EAE via inhibiting the infiltration of encephalitogenic T cells and activated macrophages and preventing ONOO^−^ - mediated neurotoxicity.

## Materials and methods

### Reagents

*Radix Rehmanniae* was purchased from KANG MEI Pharmaceutical Co., Ltd (Guangdong, China). Mouse myelin oligodendrocytes glycoprotein (35-55) peptide (MOG_35−55_, MEVGWYRSPFSRVVHLYRNGK) with the purity of over 96% (wt/wt) was purchased from Chinese Peptide Company (Zhejiang, China), incomplete Freund's adjuvant from Sigma-Aldrich (St. Louis, MO, USA), Mycobacterium tuberculosis H37RA from BD Biosciences (Difco, BD) and *Pertussis Toxin* from List Biological Laboratories (CA, USA). Percoll gradient was purchased from GE Healthcare Life Sciences (Pittsburgh, PA, USA). Cell surface-staining antibodies obtained from eBioscience (San Diego, CA, USA), including CD45-PE (30-F11), CD3e-FITC (145-2C11), CD4-Pacific Blue (RM4-5), and CD11b-APC (M1/70). Primary antibodies for 3-NT and iNOS were obtained from Abcam (Cambridge, UK); Bax, p-p65^Ser536^, p65, p-IKKα/β^Ser176/180^, IKKβ, p-IκBα^Ser32^, IκBα, and GAPDH from Cell signaling Technology (Beverly, USA); p47 ^phox^ and p67 ^phox^ from Santa Cruz (Dallas, TX, USA). For HPLC analysis, all solvents used were of HPLC-grade. Catalpol with over 98% purity was purchased from Shanghai Tauto Biotech. Co., Ltd (Shanghai, China). ONOO^−^ donor 3-morpholinosydnonimine (SIN-1) was purchased from Cayman Chemical (Ann Arbor, MI, USA), Lipopolysaccharides (LPS) from *Escherichia coli* O111:B4 was purchased from Sigma-Aldrich. HKYellow-AM, an ONOO^−^ selective probe, were obtained from Professor Yang Dan's laboratory (Chemical Biology, HKU, HK).

### Preparation of RR extract

The dried RR materials were cut into small pieces (about 0.2 × 0.2 × 0.2 cm). The sliced samples (400.0 g) were macerated overnight and repeatedly ultrasonic-extracted with 80% ethanol/water (3 × 4 L) for 40 min each time. Then, the extracted solutions were evaporated under vacuum (30°C) to remove ethanol and remaining aqueous were frozen and freeze-dried to obtain RR extract powder (162.8 g). The procedure of the RR extraction was restrictively standardized for the quality consistence.

### Qualitative analysis

To characterize the chemical profile of RR, LCMS-IT-TOF (Shimadzu, Kyoto, Japan) was adopted. The system equipped with a SIL-20AC auto-injector, two LC-20AD pumps, a CTO-20A column oven, a SPD-M20A DAD and an electrospray ionization (ESI) interface. Mass spectrometric analysis was performed with QIT coupled to TOF mass spectrometer.

Chromatographic separations were achieved on an AQ-C18 column (5 μm, 4.6 × 250 mm, ACE, Scotland). The chromatographic conditions were as follows: flow rate of 0.8 mL/min, sample injection volume of 10 μL, column temperature of 25°C and mobile phase A (0.1% formic acid-water) and mobile phase B (acetonitrile). The gradient profile was optimized as the following: 0–10 min, 1% B; 10–20 min, 1–2% B; 20–25 min, 2–5% B; 25–55 min, 5–15% B; 55–65 min, 15–25% B; 65–80 min, 25–45% B; 80–85 min, 45–70% B; 85–90 min, 70–90% B.

The electrospray source of the MS was operated in positive/negative ion modes and the operating parameters were: nebulizing gas flow rate, 1.5 L/min; the heated capillary temperature, 200°C; CDL temperature, 200°C; capillary voltage, 4,000 V; detector TOF voltage, 1,600 V. Full scan mass spectra were acquired from m/z 100 to m/z 1,000 Da with accurate mass measurement of all mass peaks. The data were processed and analyzed by LCMS solution Software Version 3.0 (Shimadzu, Kyoto, Japan).

### Quantitative analysis

Catalpol has been reported to ameliorate the pathological process of EAE mice (Yang et al., 2017; Li et al., 2018). Thus, we selected Catalpol as mark compound for quantitative quality control of RR. Catalpol was determined at 210 nm wavelength using UHPLC UltiMate 3000 (Thermo Fisher Scientific, USA). The chromatographic conditions were the same with qualitative experiment described as previous. RR extract powder was accurately weighed, dissolved in 70% MeOH by sonication and filtrated through 0.45 μm filter for quantitative analysis.

For validation of the quantitative methodology, linearity, sensitivity, precision, accuracy, and stability were detected as previous described (Liu et al., 2013). Briefly, stock solution (1 mg/mL) of Catalpol was prepared in 50% MeOH. Six concentrations of Catalpol stander were analyzed in triplicates in HPLC to prepare calibration curves. Accuracy and precision were evaluated by measuring the intra-day and inter-day variabilities and recovery of standard compounds. Stability was conducted by analyzing RR extract over a period of 2, 4, 6, 8, 12, and 24 h. The limits of detection (LOD) and limits of quantitation (LOQ) under the present conditions were determined at an S/N (signal/noise) of about 3 and10, respectively. The data were monitored, recorded and analyzed by Chromeleon Version 7.2 (Thermo, USA).

### Animals

Female C57BL/6N mice (8–10 week old) were obtained from the Laboratory Animal Unit, the University of Hong Kong. All animal care and experimental procedures were approved by the University Committee on the Use of Live Animals in Teaching and Research (CULATR). The mice were housed in the pathogen free environment with 12 h dark/light cycles.

### EAE induction and treatment

Female C57BL/6N mice were immunized for active induction of EAE as our previous described (Wu et al., 2016). Briefly, the mice were subcutaneously injected with 200 μg MOG35–55 in complete Freund's adjuvant containing 5 mg/ml heat killed *Mycobacterium tuberculosis H37RA*. *Pertussis Toxin* (200 ng) was injected intravenously twice on 0 and 2 days post-immunization (dpi). Body weights and clinical scores were measured daily. To evaluate clinical severity, EAE symptoms were scored as follows: 0, no clinical signs; 0.5, partially limp tail; 1, paralyzed tail; 1.5, hindlimb paresis or loss in coordinated movement; 2, loss in coordinated movement and hindlimb paresis; 2.5, one hindlimb paralyzed; 3, both hindlimbs paralyzed; 4, hindlimbs paralyzed, weakness in forelimbs; 5, forelimbs paralyzed.

For drug administration, the dosage of RR was determined according to the equivalent dose of mice to human subjects (60 g crude drug/ 60 kg). RR extract powder was dissolved in 0.3% (wt/vol) sodium carboxymethyl cellulose (CMC-Na) in saline. RR extract (3.7 g/kg/day) were orally administrated into the EAE mice on daily basis starting at 2 dpi for prevention protocol or 11 dpi for treatment protocol. The vehicle and normal groups were treated with an equal volume of 0.3%CMC-Na saline as control.

### Cell lines

Human neuroblastoma SH-SY5Y cells and mouse RAW264.7 macrophages were purchased from American Type Culture Collection (ATCC, Manassas, VA). Cells were cultured in high glucose Dulbecco's Modified Eagle Medium (DMEM) with 10% heat-inactivated fetal bovine serum (FBS, Gibco), 1% penicillin/streptomycin (PS, Gibco) and 1% 2 mM L-glutamine (Gibco). For subculture, SH-SY5Y cells were collected by trypsin digestion and RAW264.7cells by using sterile cell scraper (Corning, USA) and passaged at a split ratio of 1:10.

### Cellular experiments

SH-SY5Y cells were used to test the ONOO^−^ scavenging capability of RR and the cells were exposed to ONOO^−^ donor SIN-1. Briefly, the SH-SY5Y cells were seeded onto 6-well plates at a density of 5 × 10^5^ cells/well and incubated with 500 μM SIN-1 for 1 h. In the RR group, the cells were treated with 50 μg/mL of RR extract for 1 h prior to SIN-1 challenge or treated with PBS as control group.

To evaluate the anti-inflammation effects of RR, RAW264.7 macrophages were activated by lipopolysaccharide (LPS). Cells were seeded onto 6-well plates at a density of 5 × 10^5^ cells/well and challenged with LPS (1 μg/mL) for 30 min. In the RR group, the cells were pre-incubated with 50 μg/ml RR extract for 1 h prior to LPS exposure. The medium was collected as conditioned medium (CM) for the following experiments.

### MTT assay

Cell viability as detected with 3-(4,5-dimethylthiazol-2-yl)-2,5- diphenyltetrazolium bromide (MTT) assay according to the manufacturer's instruction. SH-SY5Y cells at a density of 5 × 10^4^ cells/well were cultured at 37°C under 5% CO_2_ atmosphere for 24 h and then incubated with MTT (0.5 mg/mL) for 4 h at 37°C. The medium was removed and 150 μl DMSO was added into each well. The absorbance at 490 nm was measured by Multi-plate Reader (Model 680, Bio-Rad). Cell viability was calculated by the absorbance values and normalized to control.

### ONOO^−^ assessment

HKYellow-AM is our newly developed ONOO^−^ fluorescent probe with high sensitivity and selectivity (Gong et al., 2015; Peng et al., 2016). We detected ONOO^−^ production both *in vivo* and *in vitro* experiments. For *in vivo* study, the mice at 18 dpi were intravenously injected with HKYellow-AM (10 μM, 1 mL/kg) at 15 min before sacrificed. After perfused with PBS, the fresh L4-L6 spinal cords were immediately dissected, embedded into O.T.C., cut into 30 μm sections, counterstained the nucleus with DAPI and imaged by a confocal laser scanning microscope LSM 780 at the conditions of excitation wavelength of 543 nm and emission wavelength of 567 nm. For *in vitro* study, cells were stained with 10 μM HKYellow-AM for 30 min and washed with PBS. The fluorescent images were captured by Carl Zeiss fluorescent microscope equipped with Axio Vision digital imaging system.

### Western blot analysis

The proteins of tissues or cells were extracted by using radioimmunoprecipitation assay (RIPA) buffer containing 1% proteinase and phosphatase inhibitor cocktails (Sigma-Aldrich). Protein lysates were separated by 11% sodium dodecyl sulfate-polyacrylamide (SDS-PAGE) gel electrophoresis, transferred onto polyvinylidene fluoride (PVDF) membrane, and separately immunoblotted with primary antibodies including 3-NT (1:1000), iNOS (1:1000), p47^phox^ (1:1000), p67^phox^ (1:1000), p-p65^Ser536^ (1:1000), p65 (1:1000), p-IKKα/β^Ser176/180^ (1:1000), IKKβ (1:1000), p-IκBα^Ser32^ (1:1000), IκBα (1:1000) and GAPDH (1:2000) following HRP-conjugated secondary antibodies (1:2000). The signals were detected by chemiluminescent ECL Select Kit (GE Healthcare, IL, USA), captured by Gel-Doc system (Bio-Rad, CA, USA) and analyzed by Image Lab software (Bio-Rad, CA, USA).

### Histopathology

Mice were perfused with PBS and then fixed with 4% paraformaldehyde (PFA). Isolated L4-L6 spinal cords were post-fixed in 4% PFA overnight at 4°C, dehydrated in gradient ethanol, permeabilized with xylene, embedded into paraffin and cut into 5 μm sections. Slides were stained with H&E or Luxol fast blue (LFB) for assessment of inflammation and demyelination, respectively. Inflammation and demyelination were scored as described previously (Li et al., 2010a). Briefly, inflammation was scored as follows: 0, none; 1, a few inflammatory cells; 2, organization of perivascular infiltrates; and 3, increasing severity of perivascular cuffing with extension into the adjacent tissue; Demyelination was scored as follows: 0, none; 1, rare foci; 2, a few areas of demyelination; and 3, large (confluent) areas of demyelination.

### Immunofluorescence

For immunofluorescence *in vivo*, post-fixed brains and spinal cord were immersed in 30% sucrose solution at 4°C for complete dehydration, embedded in O.C.T and cut into 30 μm sections. Sections were co-stained with primary antibodies CD3 (1:400) and CD11b (1:400). For *in vitro* immunofluorescence, cells were seeded onto 12 mm glass coverslips. After different experiment, cells were fixed in 4% PFA for 20 min and stained with primary antibodies p65 (1:400). After washed by PBS, the sections or cells were stained with fluorochrome conjugated secondary antibodies, counterstained the nucleus with DAPI and mounted with antifade medium. Immunofluorescent images were captured by a confocal laser scanning microscope LSM 800 (Carl Zeiss).

### Flow cytometry

Mice were sacrificed at 30 dpi. Spleen, brain and spinal cord tissues were dissected out, homogenized and suspended into single-cells. Mononuclear cells (MNCs) were harvested using density based Percoll gradient centrifugation. The isolated MNCs were stained with surface markers, CD45-PE, CD3e-FITC, CD4-Pacific Blue, and CD11b-Alexa700. Flow cytometric analysis was performed on FACS LSR II (BD Biosciences, CA, USA) and data were analyzed with FlowJo software (Treestar, Ashland, OR).

### Statistical analysis

Data were expressed as the Mean±SEM. Statistical analysis was assessed by using unpaired Student's *t*-test for two group designed comparisons or one-way ANOVA followed by Dunnett's multiple-comparison test for multiple group comparisons. All analyses were performed using GraphPad Prism Version 6.0 software (GraphPad Software Inc., CA, USA). *p* < 0.05 was considered as statistical significance.

## Results

### Qualitative and quantitative analysis of RR

**Figure 1:**
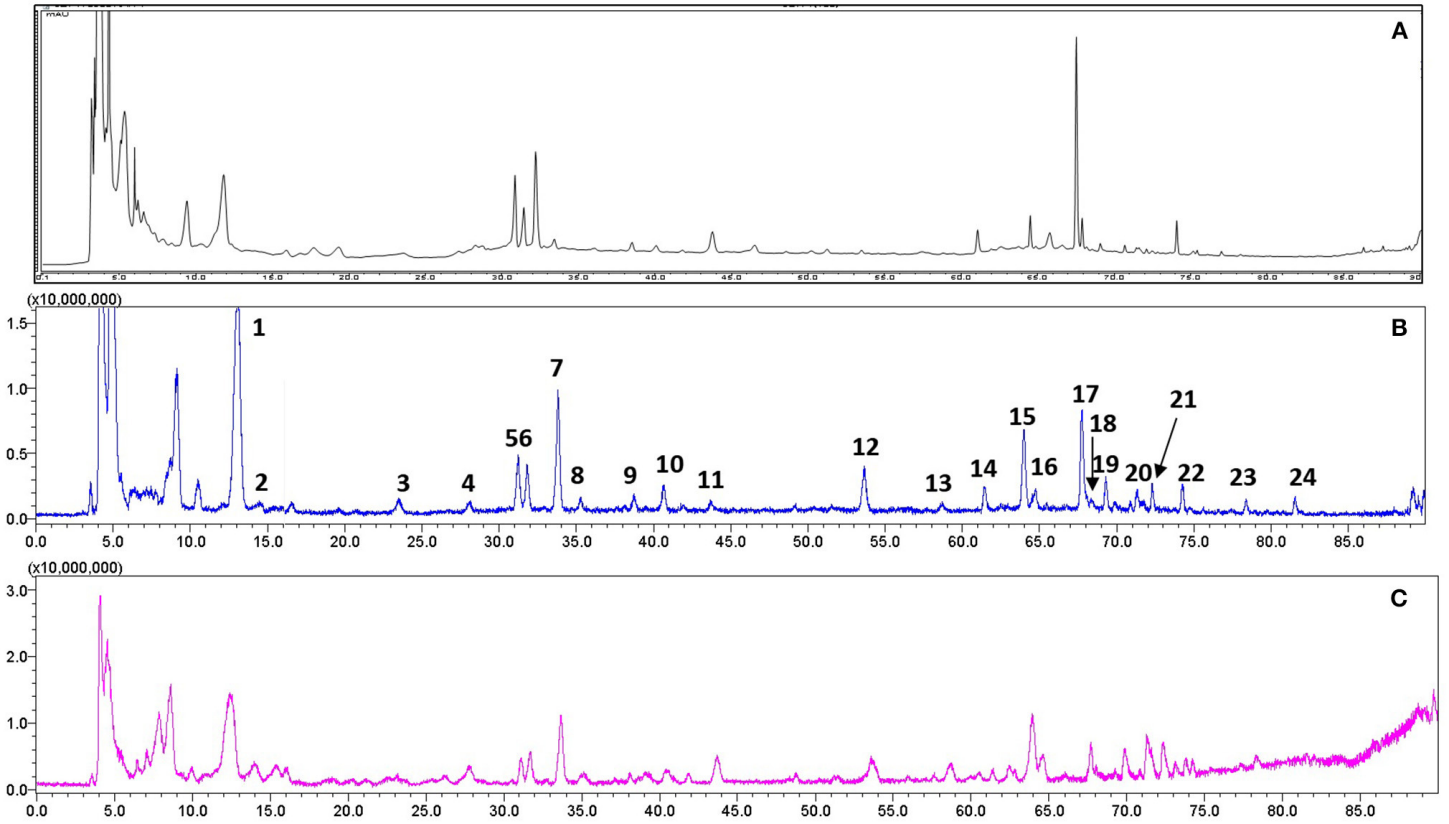
Representative chromatograms of RR extract analyzed on LCMS-IT-TOF. **(A)** UV chromatogram at 210 nm; **(B)** Total negative iron chromatogram; **(C)** Total positive iron chromatogram.

For quality control study, we identified the chemical ingredients of RR extract by LCMS-IT-TOF. Chromatographic condition was optimized and a well-separated fingerprint was obtained (Figure 1A). In both positive- and negative- ion models, a total of 24 compounds were identified by comparing their UV spectra, matching diagnostic ions and fragmentation pathways with reference compounds (Li et al., 2010b; Xu et al., 2013) (Figures 1B,C). As shown in Table 1, the structures and fragment ions of identified compounds were summarized. The identified compounds in the RR extract were iridoid glycosides and phenethylalcohol glycosides, 12 of which were iridoid glycosides (**1, 2, 4-10, 12, 15**, and **21**), 8 of which were phenethylalcohol glycosides (**11, 14, 16-20**, and **22**). Others were triterpenoids or phenolic acids.

**Table 1:** Compounds identified in RR extract.

**Peak no**.	**Retention time (min)**	**[M–H]^−^**	**[M+HCOO]^−^**	**(–) ESI-MS (m/z) Main fragment ions**	**Formula**	**Identification**
1	13.04	361.1158	407.1190	361.1139; 199.0627; 169.0500; 384.9359; 248.9609; 154.9788	C_15_H_22_O_10_	Catalpol
2	14.56	397.0922	443.0969	365.1044; 316.9483; 297.1224	C_15_H_23_ClO_10_	Glutinoside
3	23.50	397.0943	443.0945	341.1098; 316.9469; 226.9778; 112.9907	C_28_H_14_O_3_	2-(Dibenzo[ghi,mno]fluoranthen-1-ylcarbonyl) benzoic acid
4	27.99	361.1171		326.1234; 316.9459; 112.9856	C_15_H_22_O_10_	Danmelittoside
5	31.37	685.2181	731.2267	384.92297; 248.9549; 263.0779; 341.1154; 248.9606; 505.1471; 685.2161	C_27_H_42_O_20_	Rehmannioside D
6	31.69	435.2243	389.1928	319.0780; 183.1034; 215.0819; 112.9959	C_20_H_36_O_10_	Rehmaionoside A/Rehmaionoside B
7	33.70	523.1702	569.1709	523.1702; 463.1343	C_21_H_32_O_15_	Melittoside
8	35.22	345.1554		367.1360; 265.1019; 165.0924; 248.9601; 154.9789	C_16_H_26_O_8_	Rehmapicroside
9	38.68	347.1311	393.1404	347.1351; 167.0743; 149.0667; 248.9611; 154.9769; 248.9596; 154.9770; 329.1333	C_15_H_24_O_9_	Leonuride or isomer
10	40.60	373.1148		318.7710; 316.9545; 248.9636; 113.0061	C_16_H_22_O_10_	Geniposidic acid
11	43.63	461.1664		315.1091; 397.1125; 204.963; 154.9775	C_20_H_30_O_12_	Decaffeoyl-verbascoside
12	53.55	375.1295		213.0715; 169.0923; 103.9678; 131.7047; 191.0681	C_16_H_24_O_10_	8-Epiloganic acid
13	58.85	451.2147		391.0532; 293.0553; 277.0559; 226.9850; 129.1799	C_27_H_32_O_6_	28-Deoxonimbolide
14	61.54	785.2529		623.2172; 461.1685; 315.1005; 477.1517; 703.1626; 541.1231	C_35_H_46_O_20_	Purpureaside C/Echinacoside
15	64.11	389.2192	435.2237	389.2192; 161.0542; 179.0726; 248.9595; 154.9754; 316.9467; 248.9457	C_20_H_36_O_10_	Rehmaionoside A/Rehmaionoside B
16	64.78	799.2666		623.2188; 461.1619; 248.9573; 703.1678; 557.0945; 384.9435; 384.9355	C_36_H_48_O_20_	Cistanoside A/Jionoside A1/Jionoside A2
17	67.75	623.1975		461.1660; 384.9374; 315.1066; 315.1115; 248.9595; 154.9775	C_29_H_36_O_15_	Acteoside
18	68.12	813.2823		637.2318; 623.2012; 461.1617; 659.2773; 513.2349	C_37_H_50_O_20_	Jionoside B1/Jionoside B2
19	69.21	623.1973		461.1660; 384.9380; 315.1153	C_29_H_36_O_15_	Isoacteoside/Forsythoside A
20	70.89	637.2136		461.1669; 452.9183; 248.9533; 316.9483	C_30_H_38_O_15_	Jionoside D/Leucosceptoside A/Leucosceptoside
21	72.29	523.1822		452.9175; 316.9470; 248.9533; 193.0507; 162.0315; 154.9770	C_25_H_32_O_12_	6-O-E-Feruloylajugol
22	74.25	651.2309		457.1848; 384.9551; 520.9061; 383.9357; 248.9616; 313.1651; 237.1449	C_31_H_40_O_15_	Martynoside/Martynoside isomer
23	78.48	535.2542		452.9211; 349.1507; 238.7846; 248.9545; 112.9959	C_28_H_40_O_10_	Strophanthidin arabinoside
24	77.89	329.2324		313.1670; 267.1626; 211.1375; 171.0987	C_18_H_34_O_5_	Octadecenoic acid

We further quantitatively analyzed Catalpol as a bioactive compound in RR. HPLC was used to verify the terms of linearity, LOD, LOQ, precision, accuracy, and stability. The linearity of standard curve was *y* = 0.0337x+6.7212 with correlation coefficients (*r*) 0.9999. LOD and LOQ of all analytes were 0.1025 μg/mL and 0.3413 μg/mL, respectively. Precision was assayed by intra- and inter-day variations (RSDs) at three concentrations of analytes in triplicate, which were 1.02 and 2.31% respectively. The analytical method had the accuracy with the overall recovery of 97.8–101.4%. For stability assay, RSD of peak areas for Catalpol within 24 h was 0.32%. These results suggest that HPLC-DAD method has good sensitivity, accuracy, and stability. The validated HPLC-DAD method was subsequently applied to measure the content of Catalpol, with the concentration of 4.32 mg/g in RR samples.

### RR alleviates EAE progression

**Figure 2:**
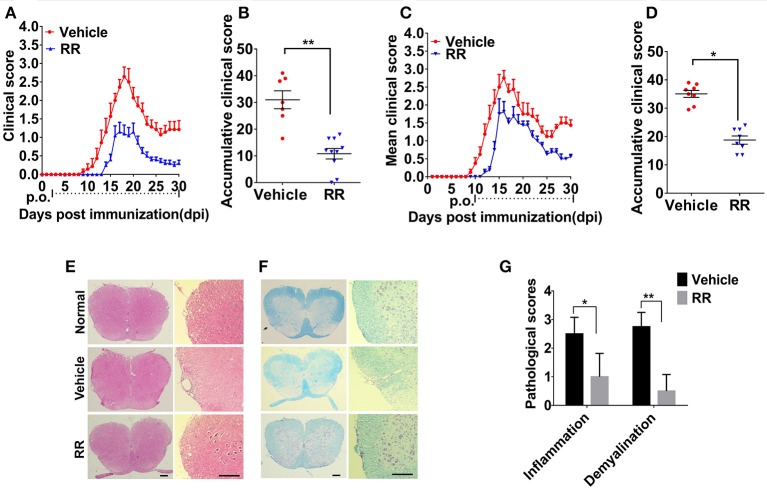
RR ameliorated active EAE via inhibiting inflammation and demyelination. Mice were administrated via p.o. with vehicle (*n* = 8) or RR (*n* = 8–10) daily starting from day of EAE induction **(A)** or disease onset **(C)**. Accumulative clinical scores of prevention protocol **(B)** or treatment protocol **(D)** for up till 30 dpi were calculated. Spinal cords from normal mice or EAE mice treated with vehicle or RR were obtained at 30 dpi (treatment protocol) and stained by H&E **(E)** and Luxol Fast Blue (LFB) **(F)** (scale bar, 100 μm). **(G)** Pathology scores of inflammation and demyelination are expressed as mean ± SEM (*n* = 4), *p < 0.05, **p < 0.01.

We then investigated the anti-inflammatory effects of RR on the EAE model. RR extract at dosage of 3.7 g/kg/day was orally administered to the mice. For the prophylactic protocol, RR treatment was started at 2 dpi. RR treatment significantly decreased daily and accumulative clinical scores in EAE mice compared with vehicle group (*P* < 0.01, Figures 2A,B). Meanwhile, RR effectively attenuated EAE progression, reduced EAE symptom severity (*P* < 0.01) and delayed disease onset (*P* < 0.05) (Table 2). For therapeutic protocol, RR treatment was started at the time of disease onset (11 dpi). Similarly, RR treatment group showed lower daily clinical scores than vehicle control group, especially at chronic phase (25–30 dpi) (Figure 2C). Moreover, RR treatment starting at 11 dpi also showed to attenuate disease severity evidenced by the mean maximal and accumulative clinical scores (*P* < 0.05, Figure 2D, Table 2). Thus, the results indicate that RR could alleviate the disease severity and progress of EAE as a prophylactic agent or therapeutic strategy. To mimic clinical therapeutic situation, further study was conducted by using treatment protocol in following EAE experiments.

**Table 2:** Clinical features of EAE in mice in the administration of vehicle or RR.

	**Treatment protocol**			**Preventive protocol**	
**Group**	**Incidence (%)**	**Mean maximal score**	**Incidence (%)**	**Mean maximal score**	**Average day of onset**
Vehicle	100	3.38 ± 0.23	100	3.07 ± 0.189	12.29 ± 0.79
RR	100	2.44 ± 0.68^*^	90	1.72 ± 0.22^**^	14.89 ± 0.35^*^

Mean ± SEM

* P < 0.05,

** *P < 0.01*.

### RR reduces inflammation and demyelination in spinal cords of EAE mice

We then investigated the neuroprotective effects of RR on EAE-induced CNS pathology. After the EAE mice received RR with treatment protocol at 30 dpi, the sections of lumbar spinal cords were detected by H&E and LFB staining. As shown in Figures 2E,F, compared with EAE mice with vehicle treatment, the RR treated EAE mice had significant lower scores of inflammatory infiltrations and smaller demyelinating areas (*P* < 0.05, Figure 2G). The results indicate that RR could attenuate CNS demyelination and inflammation in EAE mice.

### RR decreases populations of CD3^+^ and CD11B^+^ cells in spinal cords and brains of EAE mice

**Figure 3:**
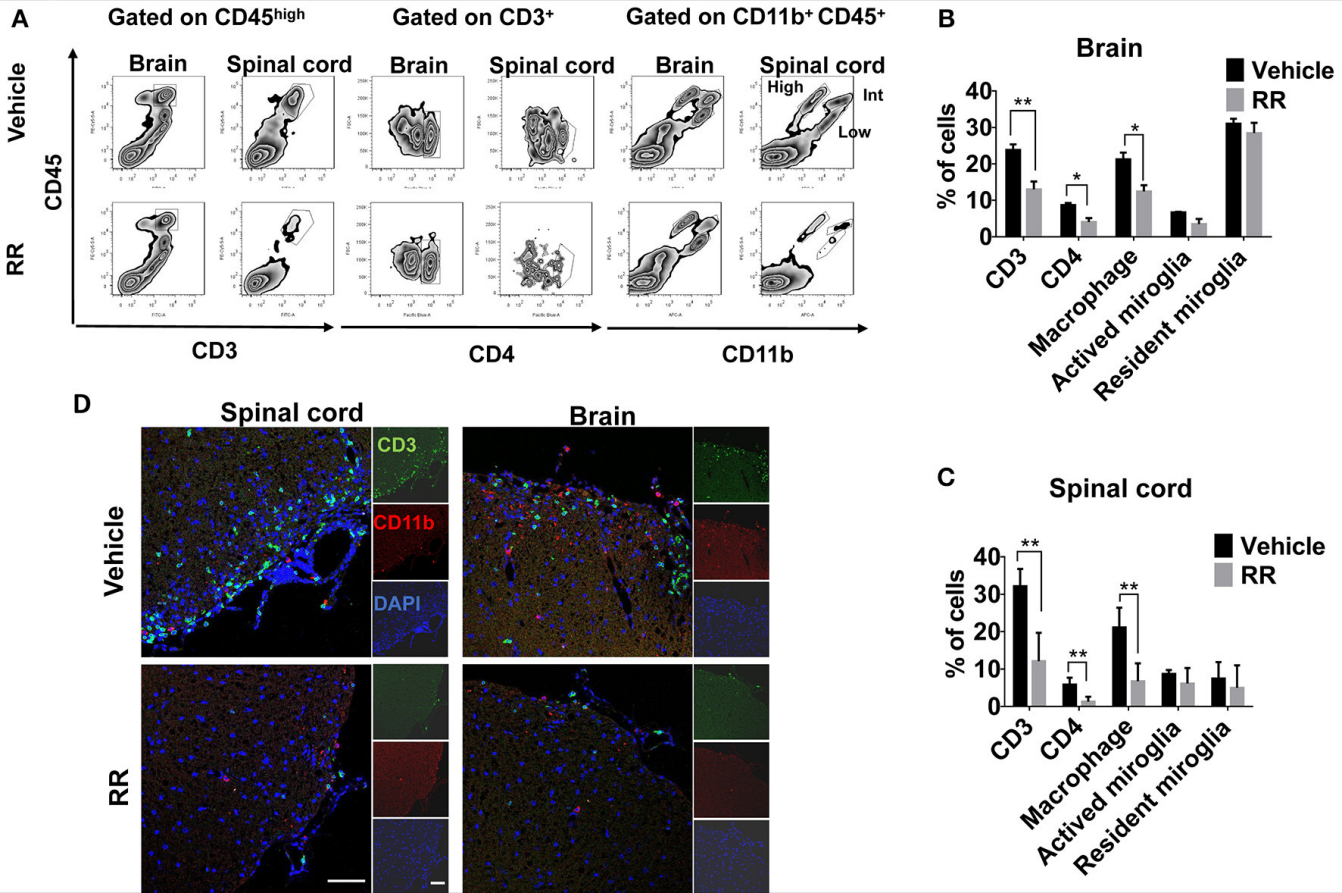
RR decreased the populations of CD3 ^+^ and CD11b ^+^ cells in active EAE. MNCs were isolated from brain or spinal cord in RR or vehicle-treated EAE mice at 30 dpi (treatment protocol). **(A)** Cells were analyzed for expression of CD3 or CD4 in the lymphocyte gate and that of CD11b in total MNC gate by flow cytometry. CD11b^+^ CD45^high^ cell was defined as macrophage, CD11b^+^ CD45^int^ as active microglia and CD11b^+^ CD45^low^ as resident microglia. Percentages of positive cells in brain **(B)** or spinal cord **(C)** are represented (*n* = 4). **(D)** Immunofluorescent co-staining of CD3^+^(green) and CD11b^+^(red) cells with nucleus (blue) in spinal cord (left) and brain (near choroid plexus within lateral ventricle, right) at 30 dpi (scale bar, 50 μm). *P < 0.05, *P < 0.01.

We next investigated different populations of the inflammatory cells between vehicle and RR treated EAE mice. Mononuclear cells (MNCs) from brains and spinal cords of EAE mice at 30 dpi (treatment protocol) were isolated and analyzed by using flow cytometry. The surface expressions of CD3, CD4, and CD11b were investigated to identify T-cell and macrophages/microglia infiltration in the lesions. Compared with vehicle EAE mice, RR treatment mice had dramatically decreased rates of CD3^+^ T cells and CD11b^+^ CD45^high^ macrophages in both spinal cord and brain tissues (*P* < 0.01, Figures 3A–C). In addition, immunofluorescence results further revealed that RR-treated mice had a smaller number of infiltrated CD3^+^ T cells and CD11b^+^ macrophages in cerebral parenchyma and the white matter of spinal cord (Figure 3D).

### RR reduces ONOO^−^ level in spinal cords of EAE mice

**Figure 4:**
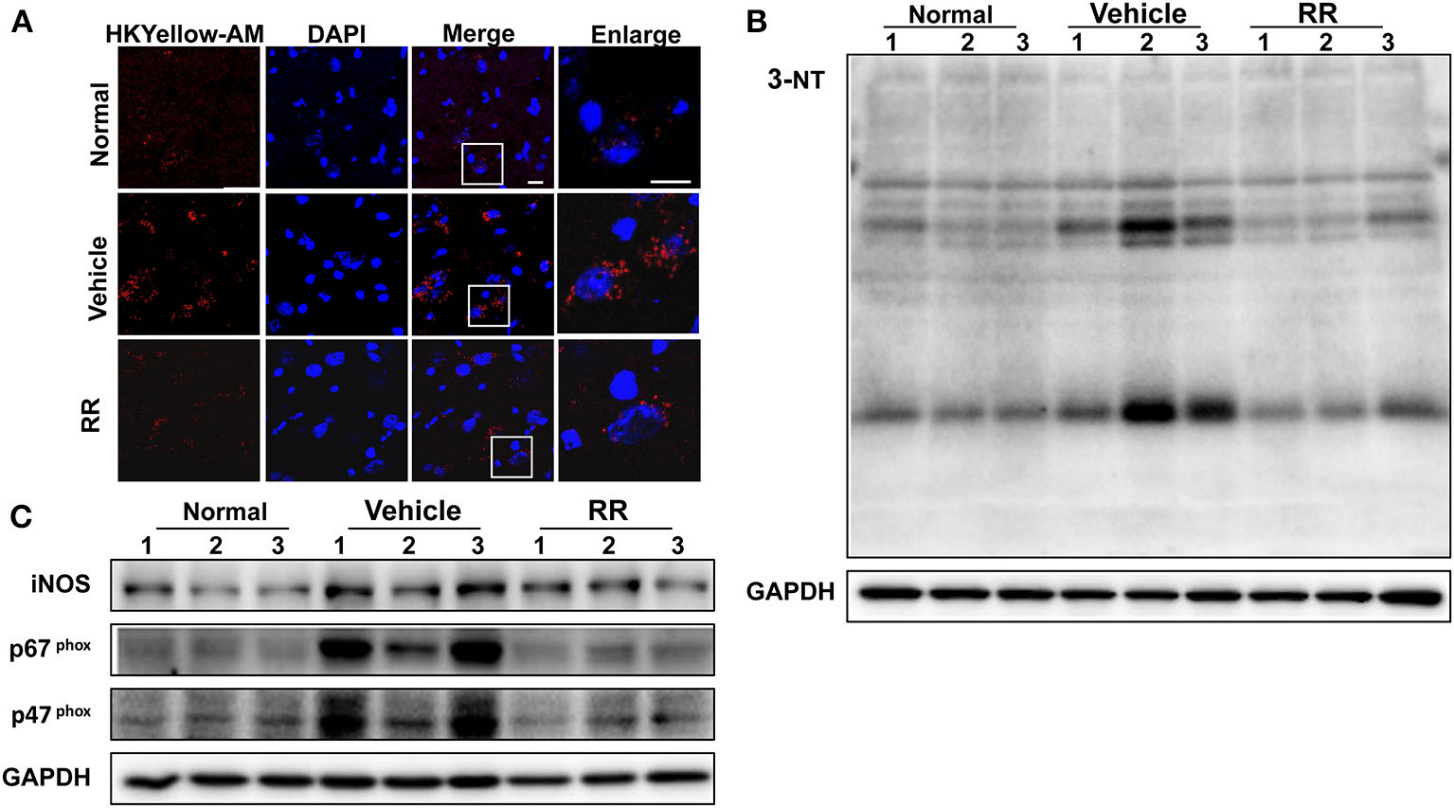
RR reduced ONOO^−^ production in active EAE spinal cord. Spinal cords in normal mice and RR or vehicle-treated EAE mice (*n* = 3) at 18 dpi (treatment protocol) were obtained. ONOO^−^ levels were detected using HKYellow-AM probe. **(A)** The immunofluorescent images of HKYellow-AM (red) and nucleus (blue) in spinal cord at 18 dpi (scale bar, 10 μm). Western blotting was conducted to analyze the expression of 3-NT **(B)** and iNOS, p67^phox^, p47^phox^ **(C)**.

We then detected the effects of RR extract on scavenging ONOO^−^ and inhibiting tyrosine nitration in the spinal cords of EAE mice by using HKYellow-AM probe and measuring 3-NT expression respectively. HKYellow-AM is a highly selective ONOO^−^ probe and used for detecting ONOO^−^ in different experimental systems (Gong et al., 2015; Peng et al., 2016). EAE mice were received RR treatment starting at 11 dpi for treatment protocol and the spinal cords were collected at 18 dpi. Fluorescent imaging study revealed that the vehicle EAE mice had remarkably increased HKYellow-AM-positive staining fluorescence, indicating the increased ONOO^−^ production in the section of the spinal cords. Compared with vehicle EAE mice, the RR treated mice showed much less ONOO^−^-positive fluorescence staining cells in the spinal cords (Figure 4A). The expression of 3-NT was also detected with western blot analysis. Consistently, EAE vehicle mice had a significantly higher expression of 3-NT in the spinal cords at 18 dpi than normal control group. The RR treatment EAE mice had remarkably decreased the expression of 3-NT in the spinal cords than the vehicle treated EAE mice (Figure 4B). In addition, the expressions of iNOS and NADPH oxidase subunit p47 ^phox^, and p67 ^phox^ were detected by using western blot analysis. The vehicle treated EAE mice had the higher expression levels of iNOS, p47 ^phox^, and p67 ^phox^ in spinal cords than the normal control mice, which were reversed in the RR treated EAE mice (Figure 4C). These results suggest that RR not only has ONOO^−^ scavenging effects but also inhibit the expression of iNOS and NADPH oxidases, subsequently reducing the production of ONOO^−^ in the EAE pathology.

### RR inhibits NF-κB signaling in the splenocytes of EAE mice

**Figure 5:**
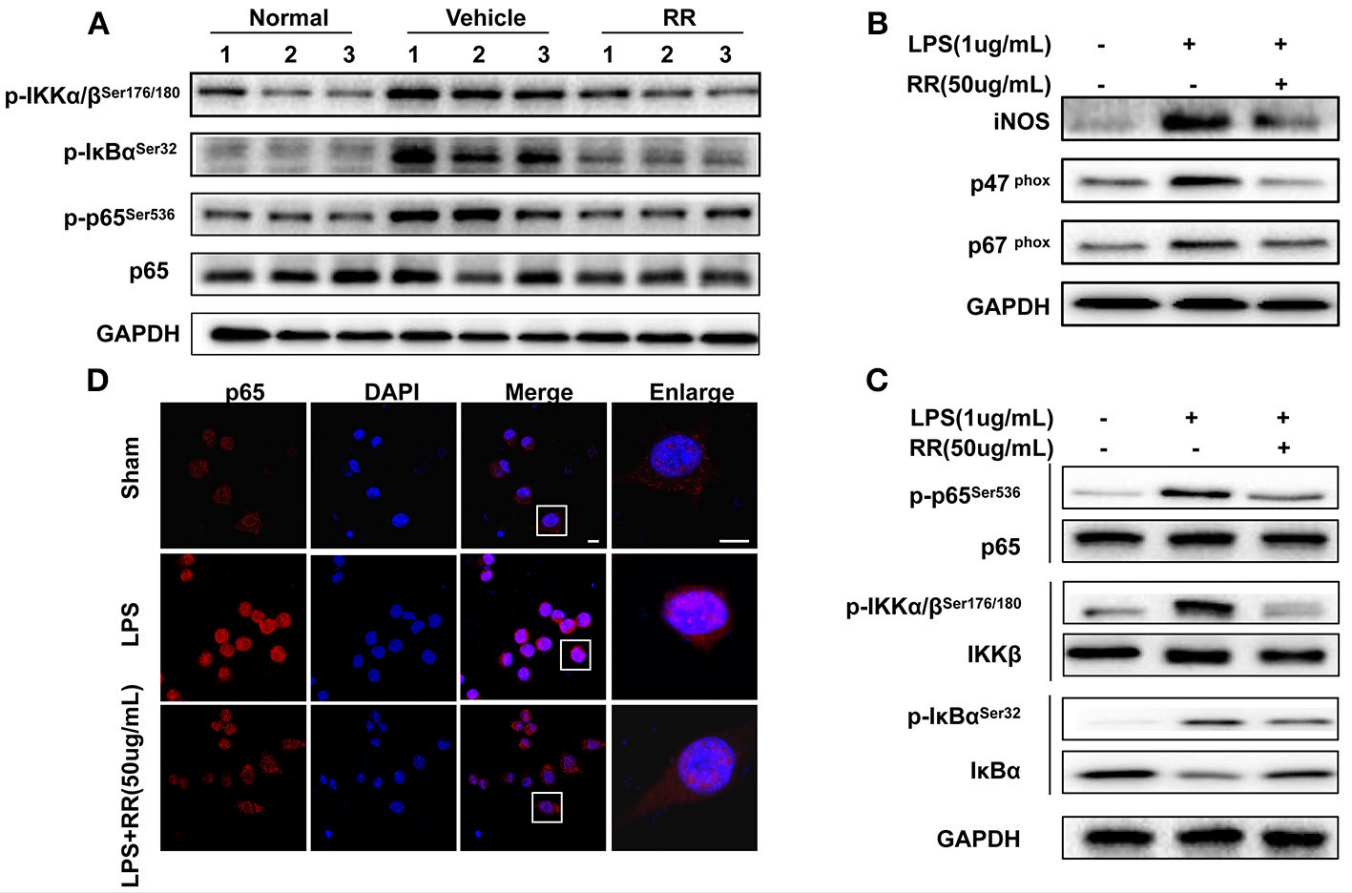
RR suppressed ONOO^−^ generation by inhibiting NF-κB signaling both *in vivo* and *in vitro*. **(A)** Splenocytes from normal, RR-, or vehicle-treated EAE mice (*n* = 3) at 18 dpi (treatment protocol) were analyzed for the expression of p65 and phosphorylated IKKα/β, IκBα and p65 by western blot assay. Macrophage cell line, RAW264.7 was pre-incubated with RR (50 ug/mL) or sham for 1 h prior to LPS (1 ug/mL) challenge for 30 min **(B–D)**. Western blotting was conducted to analyze the expressions of iNOS, p67^phox^, p47^phox^ **(B)** and NF-κB signaling-associated proteins **(C)**. The nuclear translocation of p65 was examined by immunofluorescent staining **(D)**. Scale bar represented 5 μm.

Transcription factor NF-κB is a crucial signaling regulating ROS/RNS production involved in the inflammatory process of EAE (Mc Guire et al., 2013). In MS/EAE, though activating NF-κB pathway, activated macrophages induces the production of proinflammatory cytokines, including ROS and NO (Glass et al., 2010). ROS/RNS reversely activate NF-κB pathway in the loop positive-feedback inflammatory process (Zhang et al., 2016). Thus, we detected the effects of RR regulating NF-κB signaling for suppressing macrophage-derived ROS/RNS production. Splenocytes, dominated by macrophages and lymphocyte, were isolated from the spleens of the normal, vehicle-, or RR-treated EAE mice with treatment protocol at 18 dpi. The expression of phosphorylated IKKα/β, IκBα, and p65 were detected by western blot analysis. The vehicle-treated EAE mice revealed the increased expressions of the phosphorylated IKKα/β, IκBα, and p65 in comparison with the normal control mice, which were significantly reduced in the RR treated EAE mice (Figure 5A). These results indicate that RR could inhibit NF-κB signaling in the EAE mice.

### RR inhibits expressions of INOS, NADPH oxidases, and NF-κB signaling in LPS-activated RAW264.7. macrophages

We then tested the effects of RR on the expressions of iNOS, NAPDH oxidases and NF-κB signaling in the LPS-stimulated mouse RAW264.7 macrophages. LPS activates NF-κB signaling and induces the expressions of iNOS and NADPH oxidases to generate ROS/RNS, subsequently mediating nitrative stress (Xie et al., 1994). As shown in Figure 5B, the expressions of iNOS, p47^phox^ and p67^phox^ in the macrophages were significantly up-regulated by LPS stimulation, which were reversed by RR treatment. Moreover, LPS stimulation induced the phosphorylation of IKK/IκBα/p65 but inhibited the expression of IκBα in the macrophages. RR treatment reduced the LPS-induced phosphorylation of IKK/IκBα/p65 and suppressed IκBα degradation (Figure 5C). Immunofluorescent imaging study also showed that RR treatment alleviated the LPS-induced p65 translocation into the nucleus in the RAW264.7 macrophages (Figure 5D). These results together suggest that RR could inhibit the activation of NF-κB signaling and the expressions of iNOS and NADPH oxidase in LPS-activated macrophages.

### RR has neuroprotective effects on ONOO^−^ challenged SH-SY5Y cells

**Figure 6:**
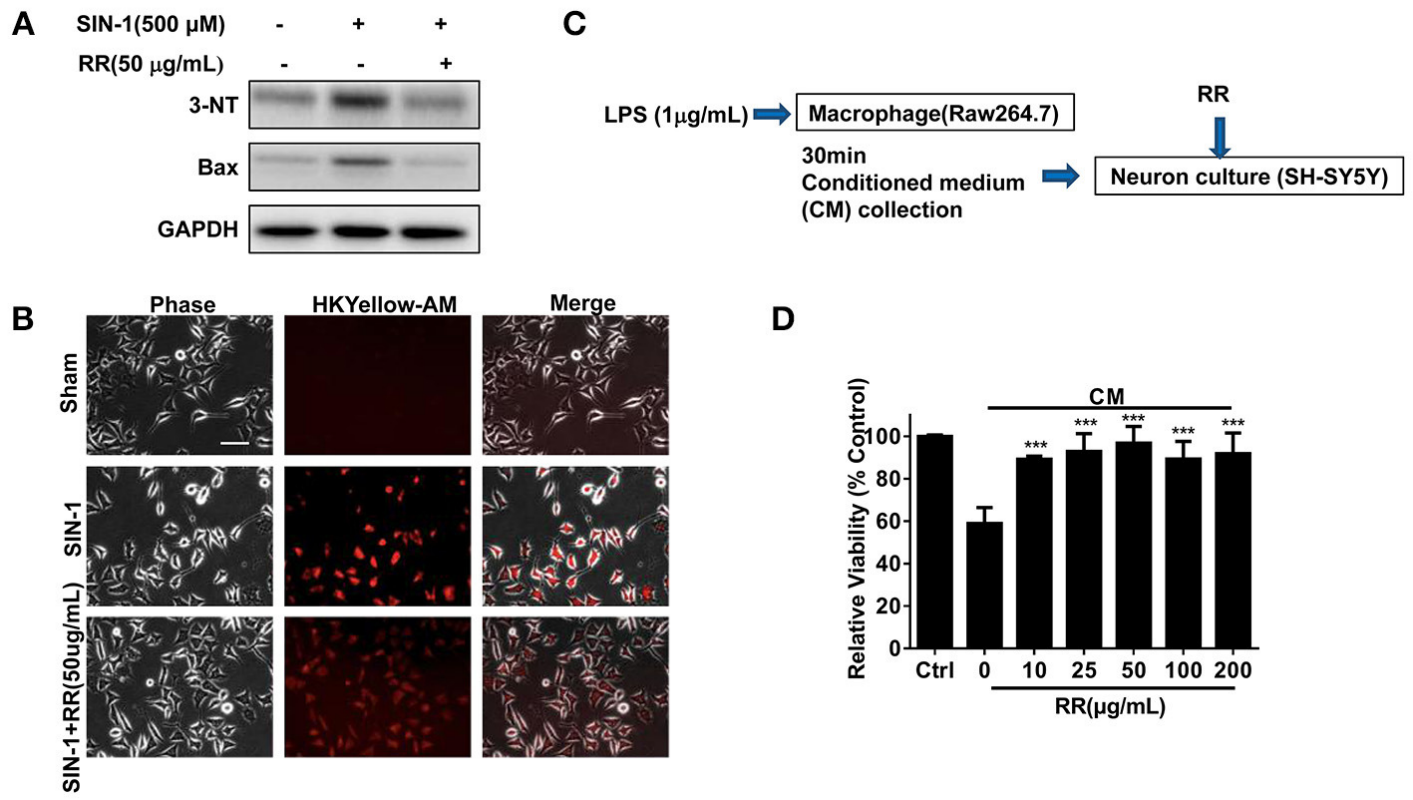
RR protected neurons from nitrative or inflammatory cytotoxicity *in vitro*. SH-SY5Y cells were pre-treated with RR extract (50 ug/mL) or sham for 1 h, followed by 500 μM ONOO^−^ donor SIN-1 for 1 h. The expressions of 3-NT and Bax were analyzed by western blot analysis **(A)**. ONOO^−^ levels in SIN-1 stimulated SH-SY5Y cells were detected using HKYellow-AM probe by immunofluorescent assay (scale bar, 20 μm) **(B)**. **(C)** The flow chart of SH-SY5Y cells under the challenge of conditioned medium (CM), mimicking inflammatory environments. Using MTT method, the viabilities of SH-SY5Y cell treated with different concentrations of RR were measured in CM or normal medium as control **(D)**. ***P < 0.001.

We finally addressed whether RR could have neuroprotective effects in the ONOO^−^ -treated SH-SY5Y cells. SIN-1, a ONOO^−^ donor, was employed to induce nitration damage on the cells. Immunoblotting analysis revealed that SIN-1 treatment up-regulated the expressions of 3-NT and Bax in the cells whereas RR treatment inhibited the increase of 3-NT and Bax (Figure 6A). Immunofluorescent study revealed that RR treatment reduced HKYellow-AM positive staining cells in the cultured SHSY5Y cells treated by SIN-1 (Figure 6B). We then investigated the effects of RR on the macrophage-derived inflammatory challenge. To mimic the inflammatory environment, conditioned medium (CM) was collected from LPS-stimulated macrophages for 30 min, which contained inflammatory cytokines or free radicals (Vijayan et al., 2017). Then, MTT assay was conducted to test cell viability in the SH-SY5Y cells treated with RR or vehicle under the incubation with conditioned or normal medium. The protocol was described in Figure 6C. As showed in Figure 6D, RR treatment dose-dependently increased the cell viability in the SH-SY5Y cells. These results suggest that RR had neuroprotective effects against ONOO^−^ induced neuronal cell death.

## Discussion

To our knowledge, this is the first report to directly demonstrate the production of ONOO^−^ in the CNS of EAE mouse model. Furthermore, for the first time, we revealed that RR could effectively suppress EAE and its underlying mechanisms are related to its anti-inflammatory and antioxidant properties via attenuating ONOO^−^ -induced nitrative stress. Our study sheds a novel insight into the underlying mechanisms of MS and provides a cue for drug discovery in the MS treatment.

Oxidative/nitrative stress is intimately associated with inflammation and immunoregulation in MS pathogenesis, resulting in demyelination, axonal degradation and neuron apoptosis (Li et al., 2011). Peroxynitrite contributes to oxidative/nitrative damage in MS pathology (Smith et al., 1999). Increased 3-NT was found in serum and CSF of MS patients (Dujmovic et al., 2009) as well as EAE mice (Bolton et al., 2008). However, with high activity and short lifespan, ONOO^−^ is difficult to be accurately detected in biological systems. Current knowledge about the roles of ONOO^−^ in MS pathology is mainly obtained from indirect evidence. It is desirable to obtain direct evidence to detect ONOO^−^ production in MS/EAE pathogenesis. In the present study, by using HKYellow-AM, we directly visualized the production of ONOO^−^ in the spinal cords of EAE mice, supporting the role of ONOO^−^ in the MS/EAE pathogenesis.

To explore the potential effects of RR against MS pathology, we made a RR extraction with restrictive protocol and conducted quality control study. We established two reliable qualitative and quantitative methods for quality control of RR by using LCMS-IT-TOF or HPLC-DAD systems. Total 24 compounds were identified. Particularly, Catalpol showed to ameliorate pathological process of EAE mice (Yang et al., 2017; Li et al., 2018). Thus, we quantitatively detected the content of Catalpol as mark compound for quality control. It is valuable to further identify other bioactive components in the RR extract related to the protection against EAE pathogenesis.

In pharmacological studies, RR extract alleviated the disease severity and progress of the EAE mice in both prophylactic and therapeutic strategy, indicating the value of RR for multiple sclerosis treatment. Previous studies showed that RR had anti-inflammatory and neuroprotective effects on systematic autoimmune diseases and its antioxidant properties might contribute to its pharmacological actions (Li et al., 2005; Tian et al., 2006). Herein, we detected the effects of RR extract on scavenging ONOO^−^ and inhibiting tyrosine nitration in the spinal cords of EAE mice by using HKYellow-AM probe and 3-NT detection respectively. RR showed its ONOO^−^ scavenging effects and inhibited the expression of 3-NT in the spinal cords of EAE mice at 18 dpi, the peak time of EAE. RR extract also inhibited the expressions of NADPH oxidase subunit p47 ^phox^, and p67 ^phox^ and iNOS in the spinal cords of the EAE mice. Meanwhile, RR attenuated ONOO^−^ -induced nitrative damage in SH-SY5Y cells treated with SIN-1, an endogenous ONOO^−^ donor via producing NO and ${\text{O}}_{2}^{•-}$ to generate ONOO^−^. In addition, to avoid the artificial results from other free radicals and observe the effects of RR extract on ONOO^−^-induced nitrative damage directly, we also conducted the experiments by using exogenous synthesized ONOO^−^ and PDC as a positive control. RR treatment inhibited the apoptosis of neurons via its ONOO^−^ scavenging properties (Supplementary Figure 1). These results indicate that RR not only have direct ONOO^−^ scavenging effects but also could suppress the production of ONOO^−^ in the EAE mice. Additionally, oxidative/nitrative stress causes mitochondrial dysfunction and amplifies the oxidative/nitrative injury (Facecchia et al., 2011). Thus, we observed the morphology of mitochondria in both *in vivo* and *in vitro* experiments (Supplementary Figure 2). RR protected from mitochondrial fragmentation in the CNS of the EAE mice and the cultured SH-SY5Y cells challenged by ONOO^−^. Thus, RR extract has strong neuroprotective effects against EAE pathogenesis possibly via scavenging ONOO^−^ and inhibiting ONOO^−^- mediated neurotoxicity.

Macrophage is the major producer of ROS/RNS, contributing to oxidative/nitrative damages in MS/EAE (Nikić et al., 2011). CD4^+^ helper T cells are crucially involved in the progressions of various autoimmune diseases including MS and EAE (Zhu et al., 2010; Mills, 2011). To investigate the anti-inflammatory effects of RR, we isolated MNCs from the brains and spinal cords of EAE mice at 30 dpi and analyzed the infiltrations of T-cells and macrophages/microglia in the lesions. RR dramatically decreased the rates of CD3^+^ T cells and CD11b^+^ CD45^high^ macrophages in the cerebral parenchyma and white matter of spinal cords in EAE mice. Meanwhile, NF-κB plays an important roles in the inflammatory and oxidative damages in EAE/MS pathology (Mc Guire et al., 2013). Activated NF-κB pathway stimulates macrophages to secret proinflammatory factors and produce ROS and RNS (Glass et al., 2010) whereas NF-κB pathway is positive-feedback modulated by ROS/RNS (Zhang et al., 2016). Our data revealed that RR inhibited the expression of the phosphorylated IKKα/β, IκBα and p65 in splenocytes isolated from the spleens of EAE mice with dominated macrophages and lymphocytes. These results suggest that RR could inhibit NF-κB signaling in immune cells of the EAE mice. The immunosuppressing effects of RR on EAE pathology are valuable to be further investigated. The potential roles of RR in modulations of immune system and the microenvironment for the release of proinflammatory cytokines could be a potential direction for further investigation.

Of note, the RR extract contains multiple ingredients in which 24 compounds were identified in this study. Among them, Catalpol is a major component identified in RR extract. Catalpol effectively inhibited NF-κB signaling, reduced NO and ROS production and attenuated LPS-induced macrophages activation and neurotoxicity in mesencephalic neuron-glia cultures (Tian et al., 2006). Catalpol attenuated cognitive impairment and protected hippocampal CA1 region neuronal cell death from oxidative/nitrative injury (Li et al., 2004, 2005). Catalpol treatment ameliorated the pathogenesis of EAE (Yang et al., 2017; Li et al., 2018). Thus, we used Catalpol as bioactive mark compound for the quality control of the RR extract. With 24 identified compounds and other potential unidentified compounds in the RR extract, we should further explore other bioactive compounds and their corresponding molecular targets contributing to the antioxidant and anti-inflammatory activities for ameliorating the pathogenesis of EAE. Importantly, given that the pathogenesis of MS involves a complex network of multiple factors or multiple signaling pathways, the neuroprotective effects of RR extract on MS/EAE pathogenesis could be achieved by the synergistic actions of multiple compounds for network regulations to the multiple molecular targets involved. Herbal medicine or TCM formulas with multiple compound may be more effective to modulate immune systems than the therapeutic strategy of single compound or drug development based on one target approach in MS treatment.

**Figure 7:**
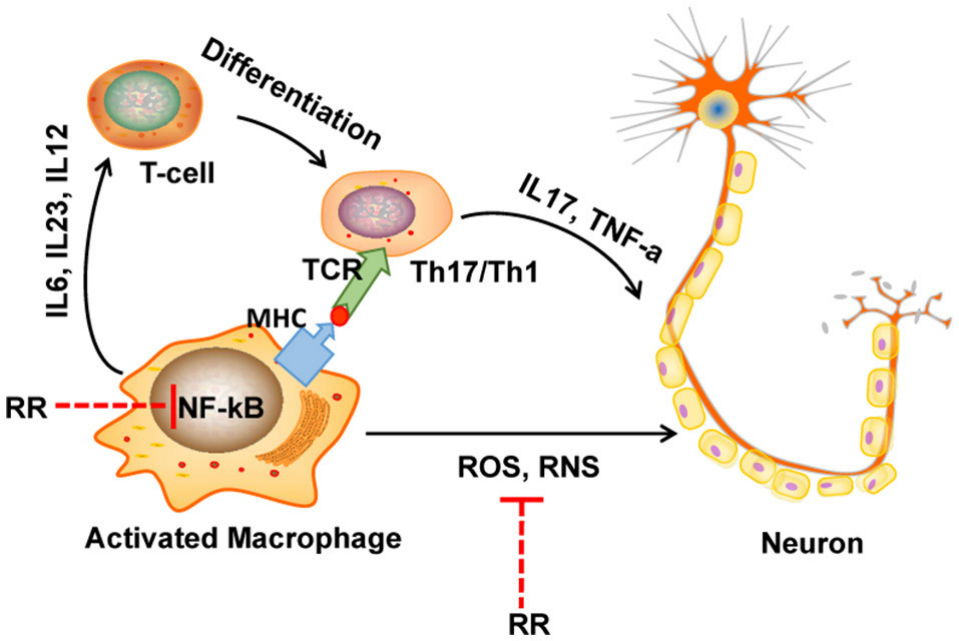
The diagram of potential mechanisms of RR in EAE treatment. In multiple sclerosis (MS), macrophage is activated by bacteria or viruses or other environmental stimuli, leading to the production of proinflammatory cytokines and free radicals via activation of NF-κB signaling pathway. Produced ROS/RNS directly destruct the structure of myelin sheath and neurons. On the one hand, in our study, RR could directly scavenge ONOO^−^, a representative RNS. On the other hand, RR also suppressed the production of ONOO^−^ though inhibiting NF-κB signaling pathway.

In conclusion, RR extract could attenuate the progress and severity of EAE through its anti-inflammatory and antioxidant effects as summarized in Figure 7. The underlying mechanisms could be attributed to the ONOO^−^ scavenging activity and the suppression of macrophages-derived nitrative stress in the CNS of EAE mice via inhibiting NF-κB signaling and iNOS, NADPH oxidase. Hence, this study provides a cue to further explore the bioactive compounds of RR and their molecular targets for MS treatment.

## Author contributions

WL performed most of the experiments, analyzed the, data and wrote the manuscript. HW helped conducting animal experiments and analyzing data, and CG assisted in capturing images. DaY developed and supplied the ONOO^−^ detection probe, HKyellow-AM. DeY contributes to the quality control study. JS designed and supervised the research, revised the manuscript, and provided funding. All authors have read and agreed with the manuscript.

### Conflict of interest statement

The authors declare that the research was conducted in the absence of any commercial or financial relationships that could be construed as a potential conflict of interest.
