# Supplementary material for: Radix Rehmanniae Extract Ameliorates Experimental Autoimmune Encephalomyelitis by Suppressing Macrophage-Derived Nitrative Damage
Source: Front Physiol. 2018 Jul 20;9:864. doi: 10.3389/fphys.2018.00864 (PMC6062770; doi:10.3389/fphys.2018.00864)
Supplement: Supplementary file 1 [file Data_Sheet_1.DOCX]

**Supplementary Fig.1**


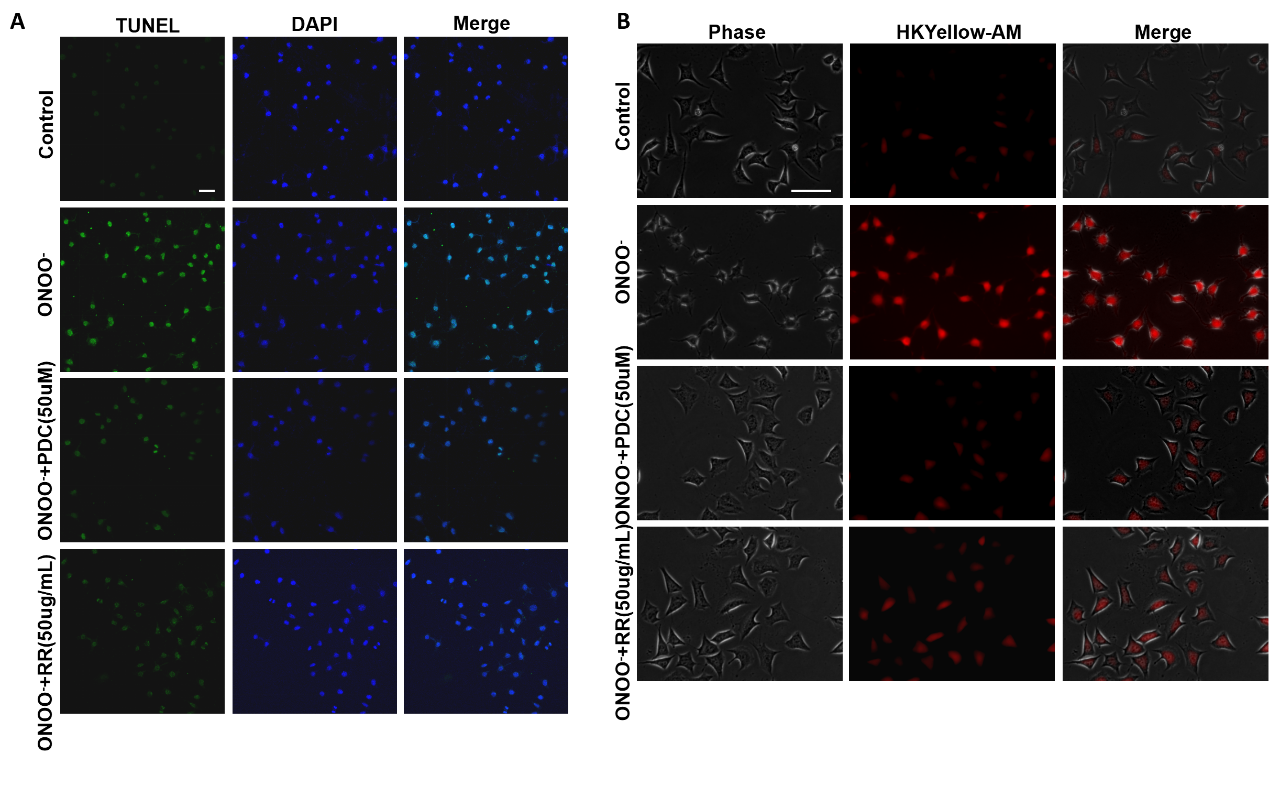


**Figure 1. RR protected neuron from nitrative cytotoxicity *in vitro*.** SH-SY5Y cells were pre-treated with RR extract (50 ug/mL), peroxynitrite decomposition catalyst (PDC) (50 uM) or sham for 1h, followed by 80 μM synthesized ONOO^-^ for 1h. Neuronal apoptosis was analyzed by TUNEL assay **(A)**. ONOO^-^ levels in synthesized ONOO^-^ stimulated SH-SY5Y cells were detected using HKYellow-AM probe by immunofluorescent assay (scale bar, 20μm) **(B)**.

**Supplementary Fig.2**


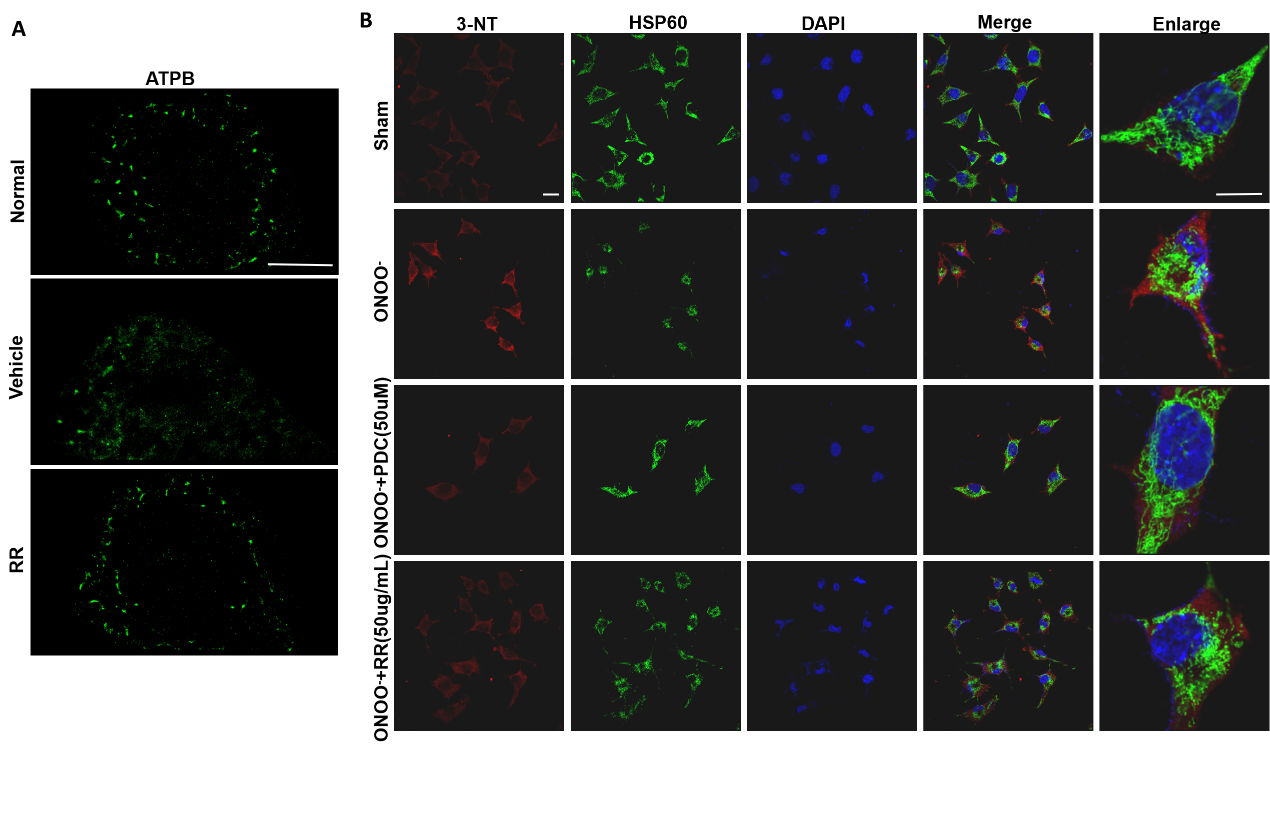


**Figure 2. RR protected neuron from ONOO^-^ -induced mitochondrial fragmentation *in vivo* and *in vitro*.** Spinal cords in normal mice and RR or vehicle-treated EAE mice at 18 dpi (treatment protocol) were obtained. Mitochondrial marker ATPB was stained and the morphology of mitochondria was examined by d-STORM immunofluorescence **(A)**. SH-SY5Y cells were pre-treated with RR extract (50 ug/mL), peroxynitrite decomposition catalyst (PDC) (50 uM) or sham for 1h, followed by 80 μM synthesized ONOO^-^ for 1h. ONOO^-^ levels and mitochondrial morphology were detected by co-staining 3-NT with mitochondrial marker HSP60(scale bar, 5μm) **(B)**.
